# Imaging analysis of 13 rare cases of renal collecting (Bellini) duct carcinoma in northern China: a case series and literature review

**DOI:** 10.1186/s12880-021-00574-8

**Published:** 2021-03-06

**Authors:** Zhehao Lyu, Lili Liu, Huimin Li, Haibo Wang, Qi Liu, Tingting Chen, Meiling Xu, Lin Tian, Peng Fu

**Affiliations:** 1 Department of Radiology, The First Affiliated Hospital of Harbin Medical University, N0.23 Post Street, Harbin, 150001 Heilongjiang People’s Republic of China; 2 Department of PET/CT, The Second Affiliated Hospital of Harbin Medical University, No.246 Xuefu Road, Harbin, 150086 Heilongjiang People’s Republic of China; 3 Department of Nuclear Medicine, Inner Mongolia Autonomous Region People’s Hospital, No.20 Zhaowuda Road, Hohhot, 010017 People’s Republic of China; 4 Department of CT, The Second Affiliated Hospital of Harbin Medical University, No.246 Xuefu Road, Harbin, 150086 Heilongjiang People’s Republic of China; 5 Department of Pathology, The First Affiliated Hospital of Harbin Medical University, Postal street No.23, Harbin, 150001 Heilongjiang Province People’s Republic of China; 6 Department of Nuclear Medicine, The First Affiliated Hospital of Harbin Medical University, Postal street No.23, Harbin, 150001 Heilongjiang People’s Republic of China

**Keywords:** Collecting (Bellini) duct carcinoma, Kidney neoplasms, Tomography scanners, X-ray computed, Positron emission tomography computed tomography

## Abstract

**Background:**

Collecting (Bellini) duct carcinoma (CDC) is a highly malignant and rare kidney tumor. We report our 12-year experience with CDC and the results of a retrospective analysis of patients and tumor characteristics, clinical manifestations, and imaging features by computed tomography (CT), magnetic resonance imaging (MRI), and positron emission tomography (PET)/CT.

**Methods:**

Retrospective examination of tumors between January 2007 and December 2019 identified 13 cases of CDC from three medical centers in northern China. All 13 patients underwent CT scan, among which eight underwent dynamic enhanced CT scan, two underwent PET/CT scan, and one underwent magnetic resonance cholangiopancreatography (MRCP) examination. The lesions were divided into nephritis type and mass type according to the morphology of the tumors.

**Results:**

The study group included ten men and three women with an average age of 64.23 ± 10.74 years. The clinical manifestations were gross hematuria, flank pain, and waist discomfort. The mean tumor size was 8.48 ± 2.48 cm. Of the 13 cases, six (46.2%) were cortical-medullary involved type and seven (53.8%) were cortex–medullary–pelvis involved type. Eleven (84.6%) cases were nephritis type and two (15.4%) were mass type. The lesions appeared solid or complex solid and cystic on CT and MRI. The parenchymal area of the tumors showed isodensity or slightly higher density on unenhanced CT scan in the 13 cases. PET/CT in two cases showed increased radioactivity intake. Evidence of intra-abdominal metastatic disease was present on CT in nine (69.2%) cases.

**Conclusions:**

The imaging characteristics of CDC differ from those of other renal cell carcinomas. In renal tumors located in the junction zone of the renal cortex and medulla that show unclear borders, slight enhancement, and metastases in the early stage, a diagnosis of CDC needs to be considered. PET/CT provides crucial information for the diagnosis of CDC, as well as for designing treatment strategies including surgery.

## Background

Collecting duct carcinoma (CDC) is a highly malignant kidney tumor which rarely occurs in clinical practice, accounting for 1–2% of renal cell carcinomas. Most patients with CDC have distant metastasis at the time of the initial diagnosis [1, 2]. Unlike renal cell carcinoma (RCC), CDC arises from the renal medulla of Bellini tubes. The biological behavior and the morphological and functional manifestations of CDC have unique characteristics, and CDC differs significantly from other types of renal carcinoma [3–6]. Here, we performed a literature review and retrospectively analyzed the imaging findings of 13 renal CDCs to provide insight into the imaging features of the disease.

## Methods

### Patient population

Thirteen cases of CDC confirmed by surgical pathology between January 2007 and December 2019 were enrolled. There were ten men and three women with an average age of 64.23 ± 10.74 years (range 46–78 years). All 13 patients underwent computed tomography (CT); eight underwent contrast-enhanced multiphase CT scan, two underwent positron emission tomography (PET)/CT, and one underwent MRCP examination.

### Acquisition protocols

The imaging techniques varied because of the retrospective nature of the study. CT exams were performed using a Philips Brilliance 256-slice CT scanner with horizontal transposition and enhanced scanning. The scanning conditions were 120 kV, 250 mA, scan matrix 512 × 512, pitch 1.0, layer thickness 5 mm, and interval 5–10 mm; the layers were partly reconstructed using a thin layer of 1–2 mm. For enhanced scanning, a non-ionic contrast agent (300 mgI/ml) at a dose of 1.5–2.0 ml/kg was administered by intravenous injection using a high pressure syringe on the anterior elbow; the flow rate was 3 ml/s, and the cortical phase started at 25 s after the injection of the contrast agent. The substantial period started at 70 s, and the renal pelvic phase started at 150 s.

Magnetic resonance imaging (MRI) was performed using the GE Signa HDx 1.5 T superconducting magnetic resonance instrument with a body phased array coil. The parameters were as follows: fast spin echo (FSE) T2WI, chemical displacement fat suppression, no pressurized Fiesta sequence scan with a layer thickness of 6 mm and a pitch of 2 mm, TR/TE18300/910, observation field 38 cm, reconstruction matrix 256 × 192, and single-slice scanning time of 2 s.

^18^F-FDG PET/CT was performed using the US GE Discovery PET/CT Elite scanner with ^18^F-FDG radiochemical purity > 95%, fasting 6 h or more before the examination, fasting blood glucose < 11.0 mmol/l, ^18^F-FDG dose of 5.55–7.4 MBq/kg. CT images were collected after 60 min of intravenous rest; the scanning parameters were 120–140 kV, the tube current was 200–300 mA, the layer thickness was 3.75 mm, and then the emission scan was performed. The examination range was from the head to the middle part of the femur. Attenuation correction was performed by CT. The PET/CT data were reconstructed using an iterative reconstruction technique. The workstation (Xeleris) was used to display, analyze, and measure the SUVmax of the primary tumor.

### Analysis methods

Two senior radiologists with 22 and 23 years of experience read the images together and evaluated the location, contour, size, internal structure, enhancement, renal arteries, calcification, surrounding structures of the lesions, infiltration, metastasis, and PET/CT findings. Lesion size was determined by measuring the maximal diameter of each lesion on axial images. CDC is divided into simple medullary involved type, cortico-medullary involved type, and cortico-medullary-pelvis involved type according to the area involved [7]. Both readers were blinded to all clinical and pathologic findings. The pattern of enhancement was classified as homogeneous if the lesion was enhanced in a uniform manner, and heterogeneous if certain areas within the lesion were more enhanced than others. Neovascularity was defined as the presence of two or more unnamed large (> 2 mm) vessels in the perinephric space adjacent to the mass [8]. In cases of disagreement between the two readers regarding any of the features, a discussion was conducted between the two readers until a consensus was reached. Thus, a consensus was reached for all features in all patients. The surgical and pathological results were compared and the characteristics are summarized.

Lesions were divided into nephritis type and mass type according to the morphology of the tumors. Nephritis-type lesions are characterized by diffuse or localized changes in the kidney. Most of the lesions are mixed density with major low density. The renal medulla is partially blurred, unclear, and the kidney outline is not changed. The mass lesions are intrarenal masses. Unlike typical carcinoma, the tumor tends to be more clearly defined and the mass protrudes from the outline of the kidney [9].

## Results

### Clinical findings

There were 13 patients including ten men and three women with an average age of 64.23 ± 10.74 years (range 46–78 years). Only one patient was younger than 50 years. The main clinical manifestations were gross hematuria in 11 (84.6%) patients and low back pain or lumbar discomfort in six (46.2%); one patient suspected of renal tuberculosis was admitted to the hospital (Table 1). This group of 13 cases of CDC were all single disease, including eight (61.5%) in the left kidney and five (38.5%) in the right kidney.

**Table 1:** Clinical data and major imaging findings of renal collecting ductal carcinoma

Case	Gender	Age (year)	Clinical manifestations	Size (cm)	Site	Involved part	Morphology	Composition	Border	Calcification	Metastasis	Examination
1	Male	61	Gross Hematuria, Low Back Flank Pain	9.14	Left	Cortex–medullary	Nephritis type	Solid-cystic	Unclear	No	Yes	Enhanced CT
2	Male	67	Irritating dry cough, bloodshot, low fever, fatigue and night sweats	5.95	Left	Cortex–medullary	Nephritis type	Solid	Unclear	No	No	Enhanced CT
3	Female	74	Gross Hematuria	12.10	Left	Cortex–medullary–pelvis	Nephritis type	Solid	Unclear	No	Yes	Enhanced CT PET/CT
4	Female	51	Gross Hematuria	7.00	Left	Cortex–medullary–pelvis	Nephritis type	Solid	Unclear	No	Yes	Enhanced CT
5	Male	76	Gross Hematuria, Low Back Flank Pain	7.09	Right	Cortex–medullary–pelvis	Nephritis type	Solid-cystic	Unclear	No	Yes	Enhanced CT
6	Male	68	Gross Hematuria	7.32	Left	Cortex–medullary	Mass Type	Solid-cystic	Unclear	No	Yes	Enhanced CT
7	Male	74	Gross Hematuria	8.00	Right	Cortex–medullary	Nephritis type	Solid	Unclear	No	No	Enhanced CT
8	Male	60	Gross Hematuria, Low Back Flank Pain	8.00	Left	Cortex–medullary–pelvis	Nephritis type	Solid	Unclear	Yes	Yes	Enhanced CT
9	Male	73	Gross Hematuria	6.08	Right	Cortex–medullary–pelvis	Nephritis type	Solid	Unclear	No	No	Unenhanced CT
10	Male	78	Gross Hematuria、Dysuria	5.12	Left	Cortex–medullary–pelvis	Nephritis type	Solid	Unclear	No	Yes	Unenhanced CT MRCP
11	Male	53	Gross Hematuria, Low Back Flank Pain	10.00	Left	Cortex–medullary–pelvis	Nephritis type	Solid	Unclear	Yes	Yes	PET/CT
12	Male	54	Gross Hematuria, Low Back Flank Pain with Frequent Urination, Urgency	12.10	Right	Cortex–medullary	Mass Type	Solid-cystic	Clear	No	No	Unenhanced CT
13	Female	46	Low Back Flank Pain	12.40	Right	Cortex–medullary	Nephritis type	Solid-cystic	Unclear	No	Yes	Unenhanced CT

### Site and border

**Fig. 1:**
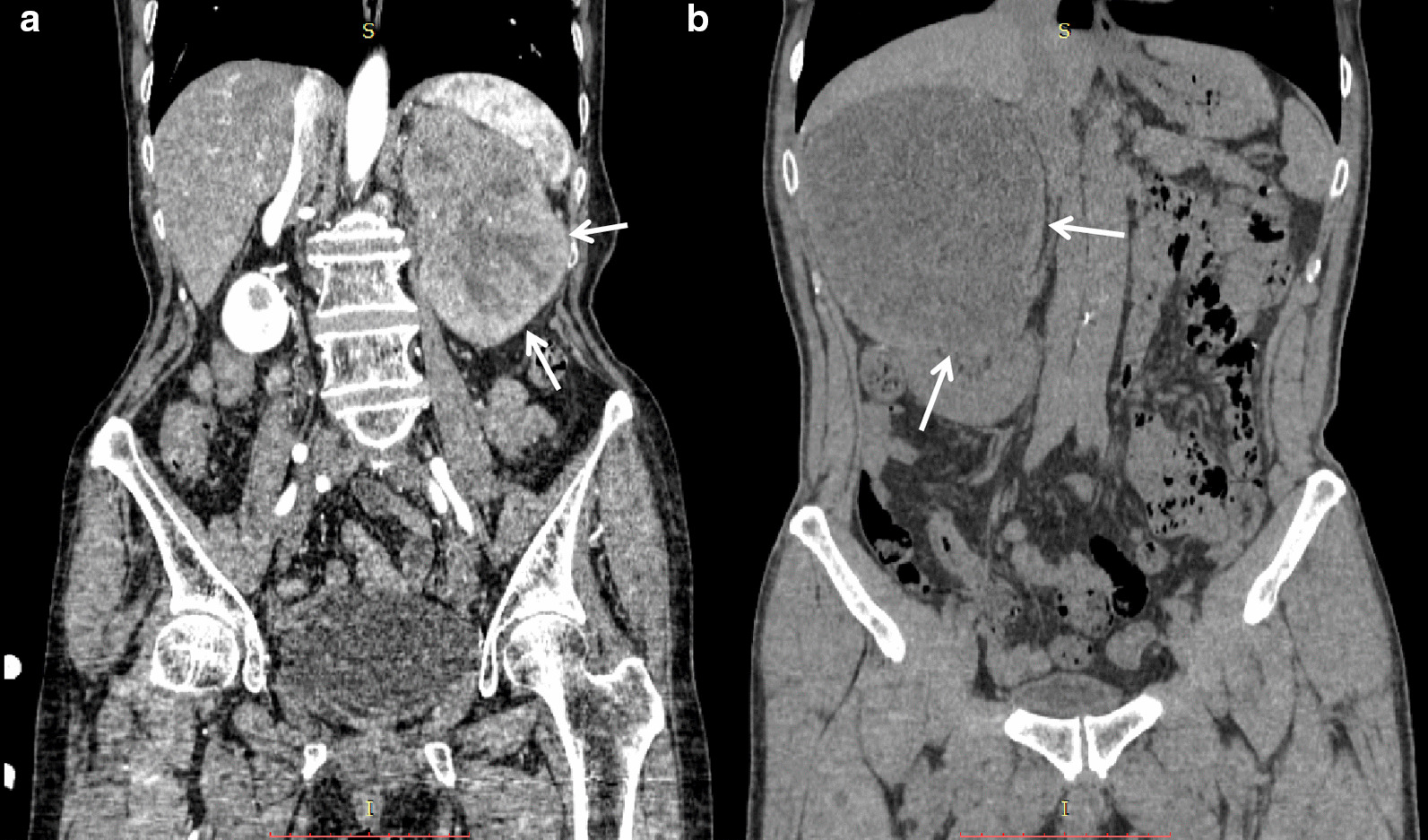
**a** Case 8: coronal enhanced CT image shows a left kidney collecting ductal carcinoma (CDC). The tumor involves the renal cortex, medulla, and renal pelvis. The boundary is unclear (arrows). **b** Case 12: coronal unenhanced CT image shows a right kidney mass-type CDC. The tumor compresses the surrounding renal cortex and medulla. The boundary is obvious, and is considered the compressed renal cortex (arrows)

The tumor site was varying, with five (38.5%) cases involved the whole kidney, five (38.5%) cases involved the upper pole of the kidney, and three (23.1%) cases involved the lower kidney. Of the 13 patients, six (46.2%) were cortical-medullary involved type and seven (53.8%) were cortex-medullary-pelvis involved type. There were no cases of simple medullary involved type (Table 1). The central part of the tumor was mostly located in the junction zone of the renal cortex and medulla, and the tumors diffused from the cortex and medulla to the inner and outer sides. The medial and renal pelvic structures were unclear. The renal pelvic and renal hilum structures were visibly damaged (Fig. 1a).

The borders of renal CDCs are mostly unclear. In this group of cases, the edge was unclear in 12 (92.3%) lesions, and no obvious signs of capsules were detected. A clear boundary was only observed in one case with a mass type lesion (Table 1). The boundary was assumed to be the compressed renal cortex (Fig. 1b).

### Morphology and size

The maximal tumor diameter ranged from 5.12 to 12.40 cm (mean diameter, 8.48 ± 2.48 cm). The diameter was > 10 cm in four (30.8%) cases and < 10 cm in nine (69.2%) cases (Table 1).

Of the 13 cases, 11 (84.6%) were nephritis type and two (15.4%) were mass type (Table 1). There were no significant increases in renal volume in three (23.1%) cases of nephritis type, eight (61.5%) cases of nephritis type had a slight increase in kidney volume, six (46.2%) cases showed a clear outline of the kidney (Fig. 1a), and two (15.4%) cases showed a lobular change in the renal contour. Two cases showed a mass-like enlargement and a marked change in kidney morphology (Fig. 1b).

### CT and MRI characteristics

**Fig. 2:**
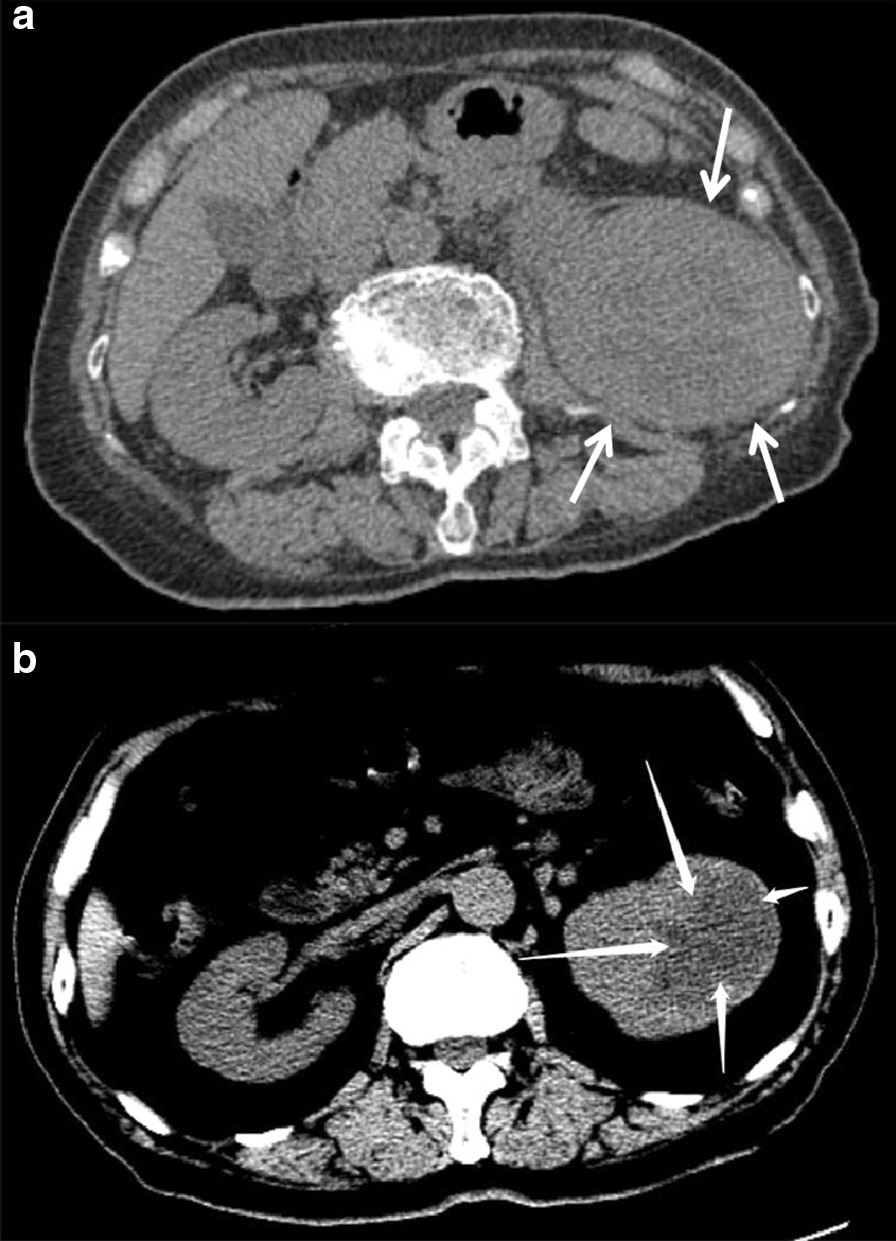
**a** Case 3: axial unenhanced CT image shows a significant increase in the volume of the left kidney CDC. The left medulla is unclear. The lesions are mainly solid components (arrows). **b** Case 6: axial unenhanced CT image shows mixed density in the left kidney and a clear cystic low-density lesion with a clear border (arrows)

**Fig. 3:**
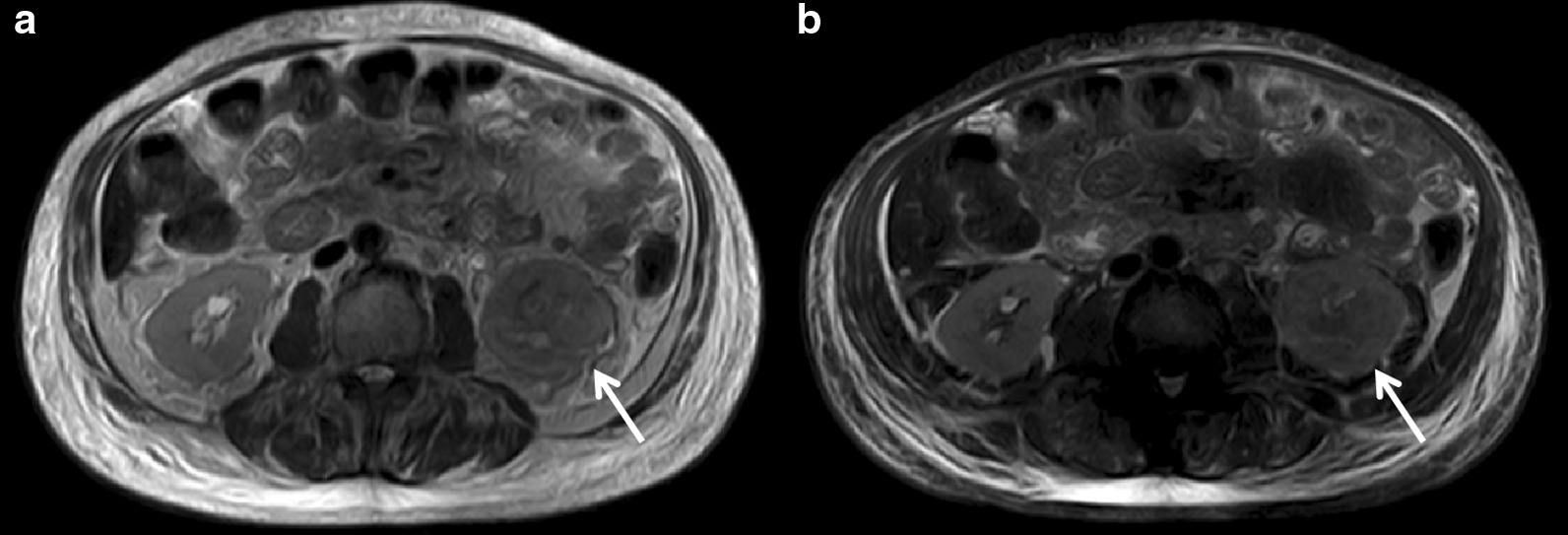
**a** Case 10: axial unenhanced T2WI image shows that the left kidney CDC signal is lower than that of the normal renal parenchyma. The renal pelvis and renal medulla are unclear (arrow). **b** Axial unenhanced fat-saturated T2WI image shows a slightly lower signal than that of the normal renal parenchyma (arrow)

The lesions appeared solid or complex solid and cystic on CT. The unenhanced CT showed high solidity (Fig. 2a), and eight (61.5%) cases had a small cystic component that represented areas of necrosis. The solid areas of the tumor were detected as soft-tissue density that was higher than that of the normal renal parenchyma. The remaining five (38.5%) lesions were complex solid and cystic (Fig. 2b), and all were single cysts. The density was uneven in 12 (92.3%) cases. Multiple sand-like calcifications were observed in one lesion. In this group, seven (53.8%) cases of cortical-medullary-pelvis involved type and two (15.4%) cases of cortex-medullary involved type showed different degrees of caliectasis. Four (30.8%) cases of cortex-medullary involved type did not show caliectasis (Table 1). On MRI, T2WI showed that the affected side was hypointense or isointense to the contralateral renal parenchyma (Fig. 3a, b). The cystic area showed a high signal intensity on T2WI.

### Enhancement characteristics

**Fig. 4:**
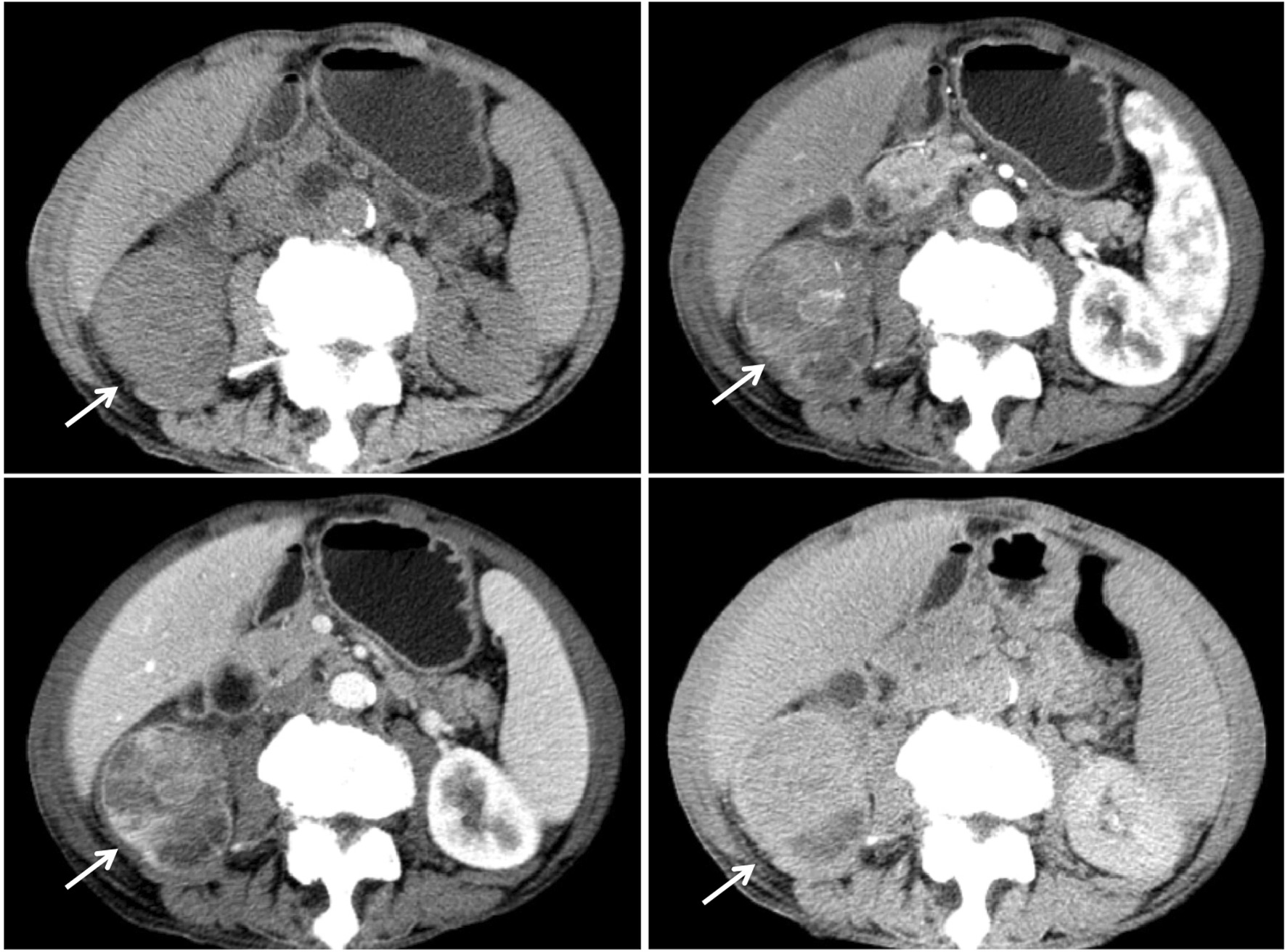
Case 5: axial unenhanced/enhanced CT image of the CDC shows that the right renal medulla is unclear. The internal density is uneven with light-moderate delayed enhancement (arrows)

Compared with the adjacent normal renal parenchyma, the parenchymal area of 13 CDC tumors was characterized by isodensity or slightly higher density on unenhanced CT. The Hounsfield unit (HU) values on CT ranged from 37.00 to 41.55 HU, and the average value was 38.71 ± 1.53 HU (the average CT value of the same layer of renal cortex was 38.17 ± 2.78 HU). In eight (61.5%) cases, uneven patchy slight (seven cases, 35.8%) to moderate (1 case, 7.7%) enhancement of the solid area of the tumor was observed on enhanced CT (Fig. 4). There was no enhancement in the cystic and necrotic areas. The mean CT value was 61.61 ± 5.81 HU in the cortical phase (the average CT value of the renal parenchyma was 170.85 ± 56.48 HU). The net increase in the CT value in the cortical phase was 23.50 HU. The CT value in the medullary phase was 68.47 ± 10.64 HU (the average CT value of the renal parenchyma was 137.44 ± 34.38 HU). In the excretion period, nine tumors showed low attenuation compared with that of the normal parenchyma. The CT value in the excretion period was 65.47 ± 5.60 HU (the average CT value of the renal parenchyma was 120.25 ± 17.91 HU). The CT value increased from 10.00 to 39.04 HU with an average of 26.90 ± 8.32 HU from the unenhanced CT to the medullary phase enhanced CT (Table 2).

**Table 2:** Characteristics of unenhanced and enhanced renal collecting ductal carcinoma

Case	Unenhanced CT value (HU)	Cortical phase CT value (HU)	Net increase CT value in cortical phase (HU)	Medullary phase CT value (HU)	Net increase CT value in medullary phase (HU)	Excretion period CT value (HU)
1	37.00	52.00	15.00	72.76	35.76	68.13
2	37.50	57.83	20.33	58.48	20.98	63.97
3	37.00	60.00	23.00	47.00	10.00	55.00
4	37.80	60.76	22.96	76.84	39.04	67.45
5	37.82	61.32	23.50	70.86	33.04	62.92
6	37.34	62.80	25.46	69.32	31.98	64.22
7	40.87	66.56	25.69	79.03	38.16	67.41
8	39.79	71.62	31.83	73.47	33.68	74.69
9	41.55	–	–	–	–	–
10	38.08	–	–	–	–	–
11	40.21	–	–	–	–	–
12	38.76	–	–	–	–	–
13	39.52	–	–	–	–	–

**Fig. 5:**
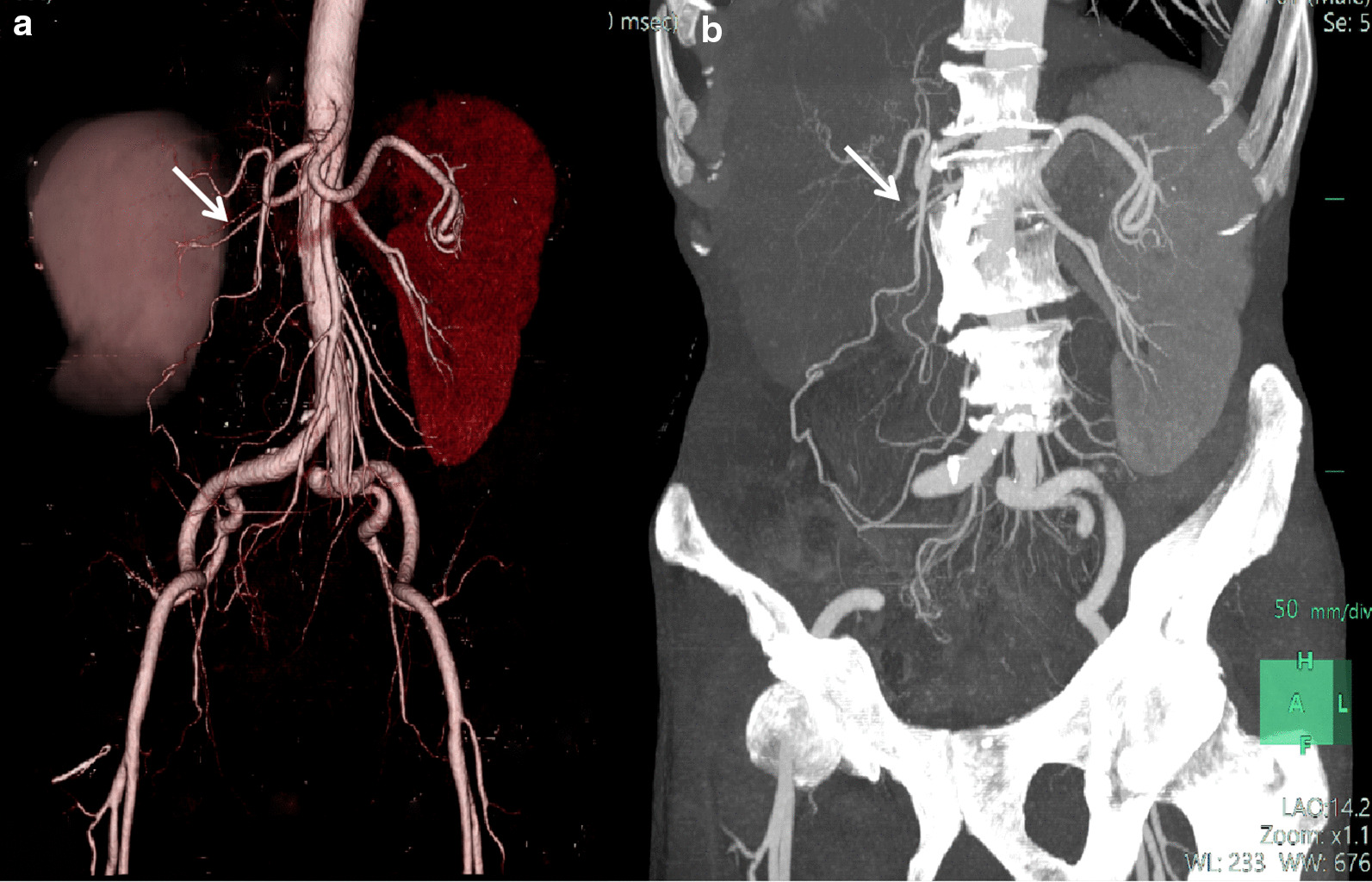
Case 5: right kidney of collecting ductal carcinoma. Volume rendering images (**a)** and maximum intensity projection images (**b**) of arterial contrast-enhanced CT show the pressure from the right renal artery (arrows)

CTA reconstruction of eight (61.5%) cases of CDC did not detect arterial enlargement or new tumor vessels. In seven (53.8%) cases, renal artery branches within tumors were attenuated or sparse (Fig. 5). Abnormal venous drainage was not observed in any of the cases.

### PET/CT findings

**Fig. 6:**
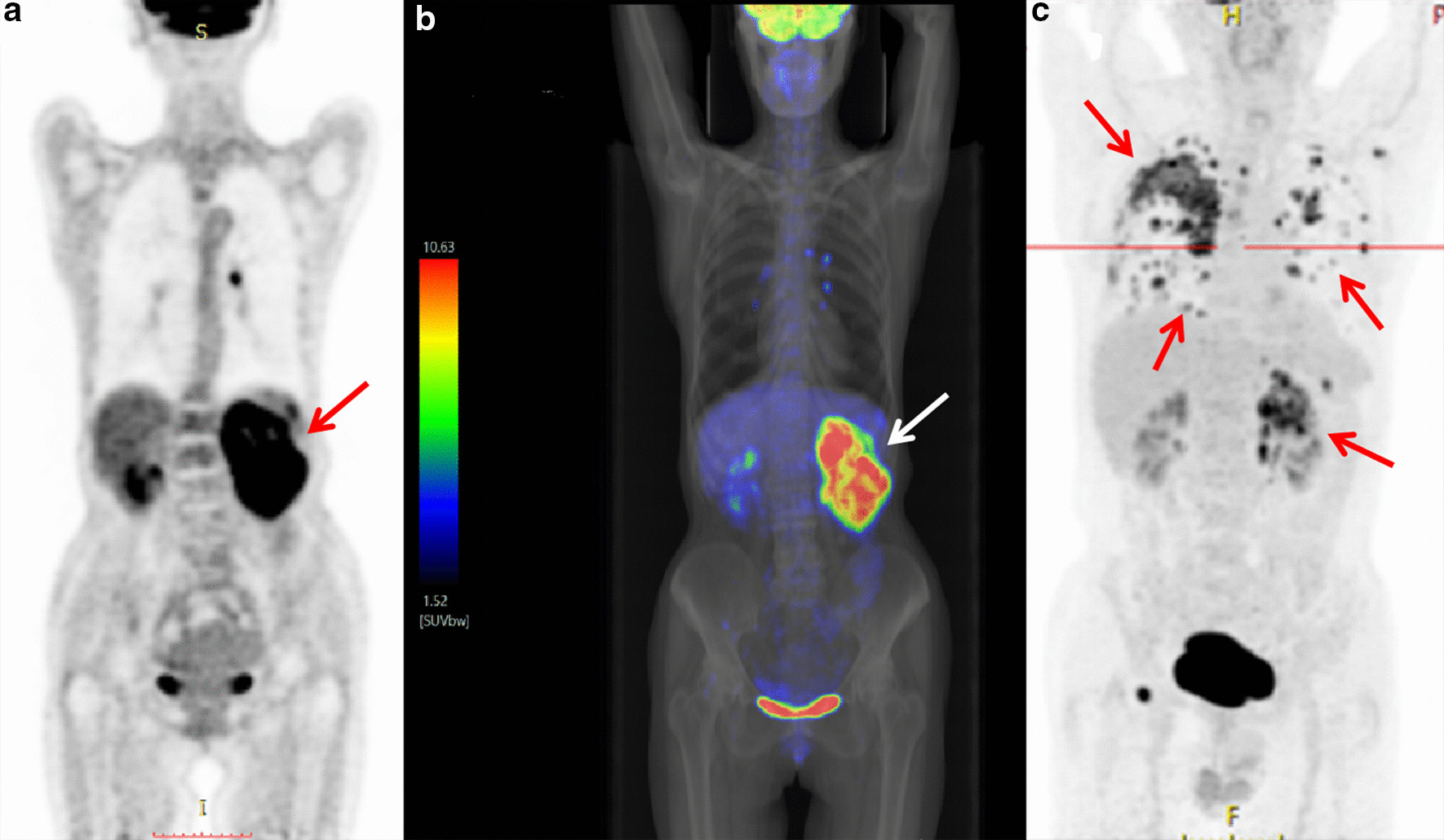
Case 11: **a** PET/CT images of renal CDC show significant concentration in the left kidney area with a maximum SUV of 14.9 (arrow). **b** PET/CT and simulated X-ray fusion images show significant concentration in the left kidney region with a maximum SUV of 14.9 (arrow). **c** Case 3: PET/CT image shows increased metabolism of the left renal CDC with extensive systemic metastases including mediastinal, retroperitoneal lymph node metastasis, homolateral adrenal gland, bilateral lungs, bilateral pleura, right ribs, left scapula, and right side pubic symphysis (arrows)

PET/CT detected increased renal radioactivity intake in two patients (Fig. 6a–c). The maximum SUV values were 14.9 and 14.3 (the maximum SUV values of the normal renal parenchyma were 3.6 and 2.9). CT detected the corresponding sites as isodense masses. In one case, the CT scan showed an unclear lesion. However, PET/CT showed significant metabolic activity with extensive systemic metastases, including mediastinal, retroperitoneal lymph node, homolateral adrenal gland, bilateral lungs, bilateral pleura, right ribs, left scapula, and right pubic symphysis metastases.

### Local invasion and distant metastasis

**Fig. 7:**
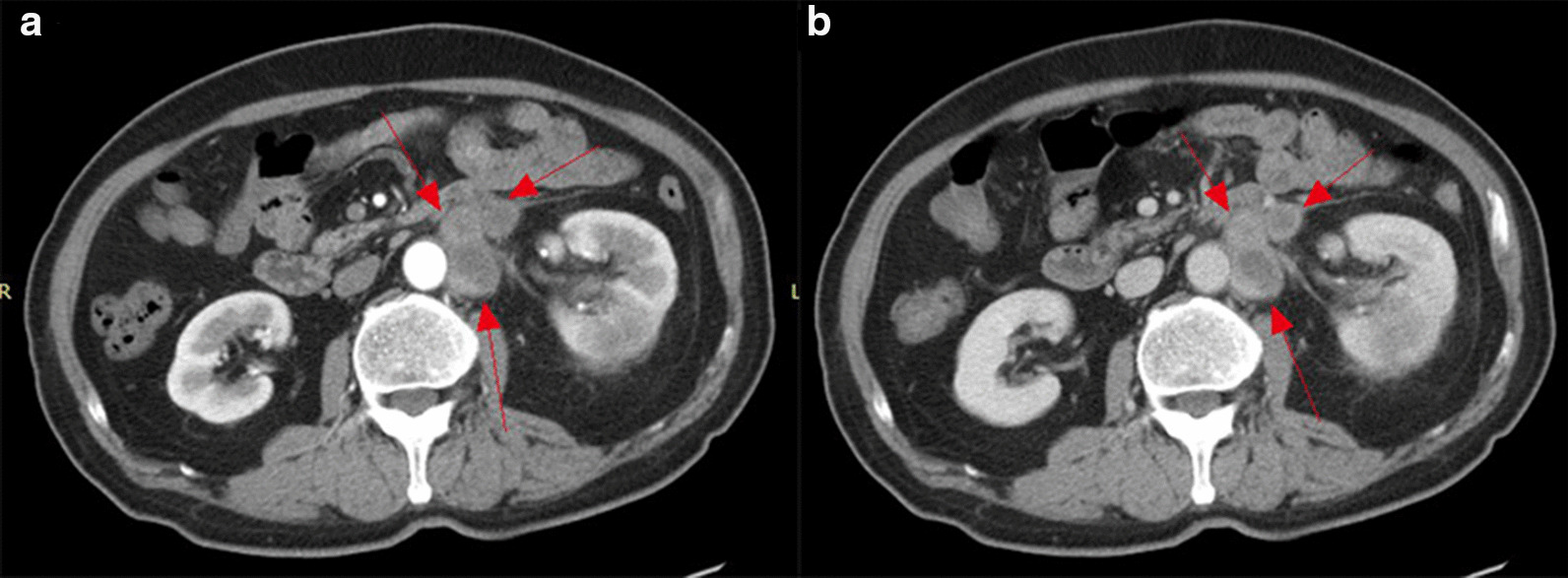
Case 6: **a** axial enhanced CT image shows left renal CDC. Axial arterial phase image showing multiple retroperitoneal enlarged lymph nodes with marginal mild enhancement (arrows). **b** Axial delayed phase image shows further strengthening, which is similar to renal CDC (arrows)

In nine of thirteen (69.23%) cases, CT images showed evidence of intra-abdominal metastatic disease (Table 1). Perirenal infiltration is common in renal CDC. In this group, perirenal fat blurring or prerenal fascia thickening was observed in 12 (92.3%) cases. Direct invasion as peritoneal and lymphatic metastasis was observed in nine (69.2%) cases (Fig. 7a, b). In seven cases, the metastases surrounded the renal artery and caused renal artery stenosis (Fig. 5a, b). Adrenal gland involvement was observed in two (15.4%) cases and included the left side in one case and bilateral involvement in one case. Inferior vena cava involvement was observed in one (7.7%) case. There were three (23.1%) cases of bilateral lung metastasis, one (7.7%) case of bilateral pleural metastasis, one (7.7%) case of brain metastasis, and one (7.7%) case of bone metastasis (right ribs, right pubic symphysis, and left scapula).

## Discussion

### Overview

**Fig. 8:**
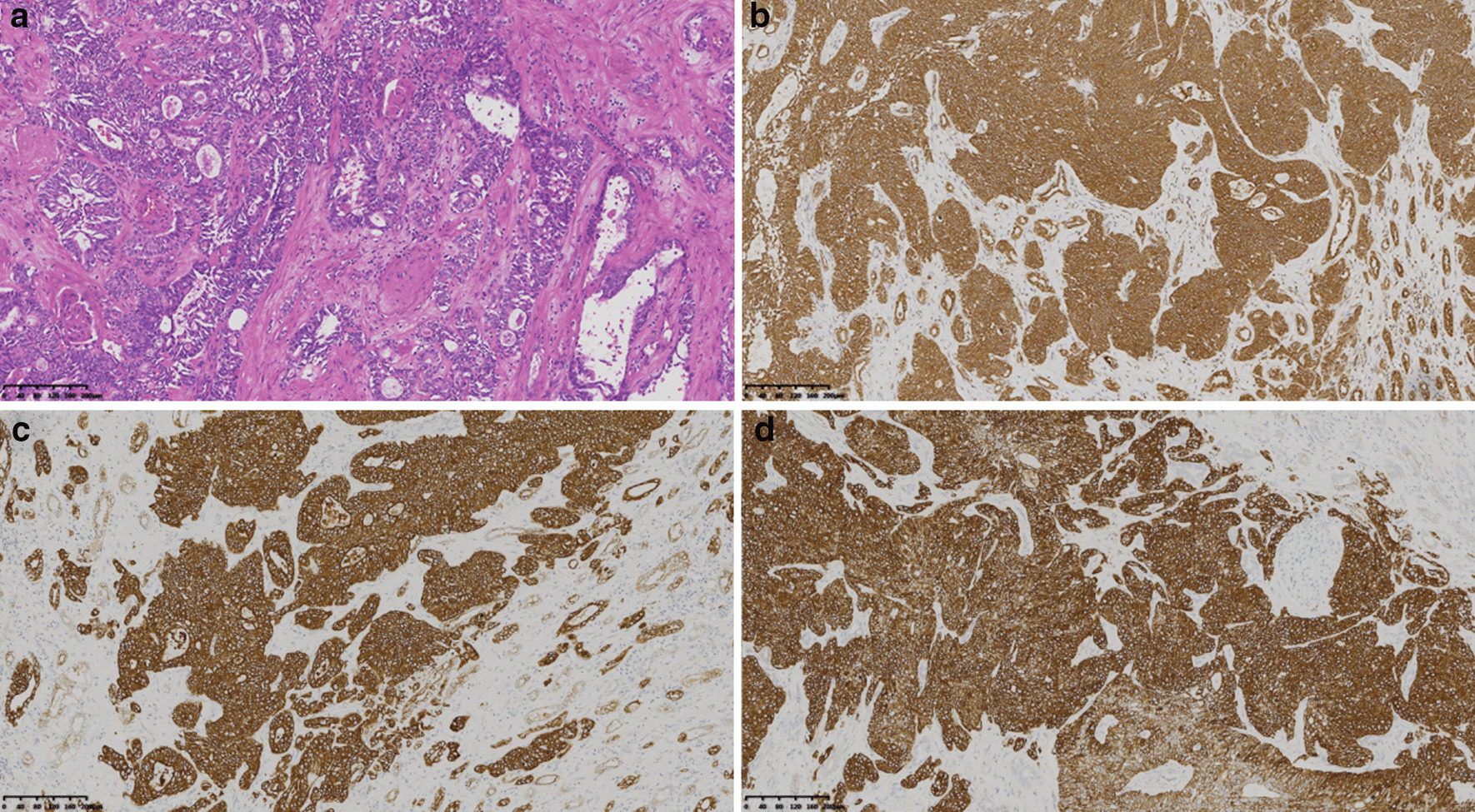
Case 1: pathological images of CDC. **a** The tumor cells are cuboidal. The cytoplasm is eosinophilic or suspicious. The nucleus is large. The nucleolus is obvious and is arranged in a small tubular or papillary pattern, and the atypical shape is obvious. Immunohistochemistry shows **b** cam5. 2(+), **c** CK(+), and **d** 34βE12(+)

In 1976, Mancilla Jimenez et al. first reported that certain papillary renal cell carcinomas originate from the collecting duct. Fleming and Lewi described six cases of CDC and presented diagnostic criteria to recognize it as a unique pathological subtype of RCC [10]. CDC was listed as one of the major subtypes of RCC in both the 2002 and 2006 WHO classifications. The tumor interstitial inflammatory fibrosis and collagen secretion are obvious, and the tumor tissue is dense. The results of the pathological examination of the patients in the present study are consistent with the literature (Fig. 8a–d).

The patients in this study showed a wide age range, with a mean age at diagnosis of 64 years and a 3:1 male predominance. Although these findings are consistent with those of previous studies [11, 12], the demographic profile also applies to RCC in general and is therefore not a useful discriminator. The clinical manifestations, which were not specific to CDC and can be found in other RCCs, included gross hematuria, waist and abdomen pain, abdominal fullness, and sometimes a palpable mass [13]. Gross hematuria was observed in 11 (84.6%) patients, and six (46.2%) had low back pain or lumbar discomfort. One patient who had no clinical manifestations of urinary disease and presented with a low-grade fever and night sweats was admitted to the hospital for suspected tuberculosis.

### Site and border

The pathological features of CDC are as follows: the initial site of the tumor is the renal medulla, which is grayish white or light yellow. The renal interstitial cells function as a scaffold for tumor cells to diffuse and infiltrate along the collecting duct to the renal pelvis and renal cortex [12]. Therefore, most of the tumors occur in the medulla and infiltrate into the cortex and renal pelvis. The borders are irregular and most kidney tumors have an outward expansive growth pattern from the center. The normal renal parenchyma is displaced and bulging of the kidney contour may be present, as well as the formation of a pseudocapsule [9, 14]. Young et al. reported that sarcomatoid RCCs and CDCs are more likely to have an irregular contour and an infiltrative pattern than other RCC subtypes. When used to discriminate sarcomatoid RCC and collecting duct carcinoma from other solid renal masses, an infiltrative spread pattern has a specificity of 93% (287/308) and sensitivity of 82% (9/11), whereas an irregular contour has a specificity of 98% (303/308) and sensitivity of 64% (7/11) [15].

CDC can be divided into medullary type, cortex-medullary type and cortex-medullary-pelvis type according to the infiltration site. The present cohort did not include patients with the simple medullary type. The masses were mostly of the cortex-medullary type (six cases, 46.2%) and the cortex-medullary-pelvis type (seven cases, 53.8%). This feature is different from the common renal clear cell carcinoma derived from cortical renal tubules [7]. The common renal clear cell carcinoma is centered on the renal cortex and invades the medulla. The CDC tumor can rapidly grow toward the renal pelvis and cortex and destroy the renal pelvis and renal hilum structure, and may involve the upper ureter. Thus, assessing for the presence of an infiltrative spread pattern and an irregular contour can provide a simple, noninvasive means of discriminating CDC from other solid renal masses with a relatively high specificity, sensitivity, and negative predictive value [15].

### Morphology and size

The morphology of tumors is closely related to the biological behavior and growth pattern of tumors [14]. Despite its medullary derivation, almost all tumors exhibit focal cortical extension, and perinephric extension is also common [16]. CDC tumors spread along the collecting duct during invasive growth, and there is fibrous tissue hyperplasia in the tumor stroma [7, 11, 17]. The tumors show a diffuse enlargement that follows the kidney contour or a certain kidney segment without clear boundaries, capsule, or pseudocapsule. This characteristic was observed in 12 of the 13 cases (92.3%) in our group. In addition, two patients showed a mass-type tumor. In one case, the inflammatory fibrous tissue proliferation was not significant. The tumor outline was bound by the tumor cell aggregation area. Therefore, we believe that the morphology of CDC tumors is related to the degree of tumor interstitial fibrous tissue hyperplasia. The extent of interstitial fibrosis is negatively correlated with the tumor outline. The morphological characteristics of the tumor are helpful to distinguish it from other kidney tumors.

The diameter of the tumors ranged from 5.12 to 12.40 cm (mean diameter, 8.48 ± 2.48 cm), and four cases had a diameter > 10 cm. In general, CDCs are relatively large. In large tumors, the medullary origin can be difficult to determine [9]. Fukuya et al. described the CT findings of small tumors measuring 3–4.5 cm. These lesions were all centered in the renal medulla; four of five protruded into the central sinus, and none showed exophytic growth; the reniform contour was preserved in all cases [18].

### CT density and MRI signal

The originating organ of CDC is water-rich kidney tissue. The tumor stroma is characterized by increased fibrous tissue hyperplasia and collagenation. The density of the tumor parenchyma is higher than that of the surrounding normal tissues, which is a feature of this group of CDC tumors in non-enhanced CT. These characteristics differ from those of RCC arising from the renal cortex. The interstitial tissue is dense or collagen secretion is increased. Inflammatory fibroblastic tissue hyperplasia and abundant fibrous tissue components are characteristic of CDC tumors. These features are important for the pathological diagnosis of CDC and appear as a low signal on magnetic resonance T2WI.

The MR examinations reported by Pickhardt et al. [9] (four cases) showed that the parenchymal components of all four tumors showed equal signals on T1WI. One tumor with multiple cystic components showed a low to high T1 signal for each cyst (including water, fat, bleeding, and other signals). On T2WI, the parenchymal components of the four tumors were lower than those of the normal renal parenchyma. There absence of a low signal at the edge of tumor indicated the presence of a pseudo-envelope observed on MRI [9]. In the present study, MRI detected the tumor parenchyma as a low signal on T2W1 in one case. The area of cystic necrosis was detected as a high and low mixed signal on T2WI, with unclear separation from the surrounding normal renal parenchyma. There was no obvious ring-shaped low T2WI signal suggesting a pseudo-envelope.

The calcification in CDC tumors is probably due to the increased fibrous tissue in the tumor stroma, and calcium salts easily deposit in fibrous tissue. However, calcification was only present in two (15.4%) cases in this group. In contrast to other RCCs, calcification was also observed in only one case in the study by Seong et al. [14]. Compared with the normal renal parenchyma, which is rich in water, the tumor tissue is denser and the interstitium shows inflammatory fibrous tissue hyperplasia. The tumor parenchyma is similar, showing a high signal on T1WI and a low signal on T2WI. Kato et al. [19] described the signal intensity of CDC on T2WI as isointense or hypointense, which was thought to be due to hemosiderin deposition. Larger clear cell renal cell carcinomas tend to have a heterogeneous hyperintense signal on T2-WI, differentiating it from CDC.

Some liquid components are detected as low-density areas in tumors. The shape is very irregular and the boundary is unclear. The shape resembles a map or a lake, which is different from the necrotic morphology of common tumors. Combined with the pathological results, the diffuse patchy low-density lesions may represent the collagen denaturation zone. A cystic lesion in the present study was diagnosed as a true cyst, which is a rare presentation in CDC. Only one of 17 cases was a cystic CDC in a study by Perry et al. [9]. When the essential components and cystic components coexist, careful analysis of the characteristics of the essential component is important for the differential diagnosis.

### Enhancement characteristics

The majority of CDC tumors in the present cohort were hypovascular. Most of the CDC tumors in the dynamic enhanced scan showed a relatively low density in the renal cortex and medulla. The parenchyma of the mass was uneven, showing light to moderate enhancement in the cortical or medullary phase that was lower than that of the surrounding renal parenchyma. The medullary phase showed uneven and mildly delayed enhancement. The degree of enhancement was lower than that of the renal parenchyma, which is consistent with the results of a Chinese study [20]. This enhancement pattern differed from that of blood-rich clear cell renal cell carcinoma, renal medullary carcinoma, renal angiomyolipoma, and renal angioma [21]. Seong et al. [14]. reported that unlike more common conventional RCC, contrast-enhanced CT scans of CDCs usually show weak (69%) and heterogeneous enhancement (85%). This enhancement pattern differs from that of renal clear cell carcinoma, which shows significant enhancement in the cortical phase, with the density reaching a peak and decreasing markedly during the medullary phase. This enhancement pattern of CDC tumors also contributes to the identification of renal clear cell carcinoma. Fujimoto et al. analyzed the enhancement pattern of RCCs greater than 5 cm in diameter on contrast-enhanced helical CT. They reported that strong enhancement comparable to that of the renal cortex was detected only in conventional RCC (75%) [22]. Jeong et al. reported that conventional renal carcinomas show a stronger enhancement than nonconventional renal carcinomas in both the corticomedullary and excretory phases. Tumors with > 84 HU in the corticomedullary phase and 44 HU in the excretory phase are likely to be conventional renal carcinomas, whereas the present data showed a 23.5 HU increase in the cortical phase [23].

In present study, CTA showed that the renal artery was involved in the blood supply and the distal branches were destroyed. The filling defect was observed in the renal vein and inferior vena cava. The tumor thrombus showed an expanded shape and the degree of enhancement was similar to that of the central necrotic and the hypovascular areas. No tumor blood vessels were observed in the vicinity of the tumor, and the original renal blood vessels were not thickened or significantly displaced. These characteristics indicate that CDC tumor cells do not produce angiogenic factors, and the original renal artery branches are rarely destroyed by tumor cells. The fibrous tissue in the tumor stroma can compress the intratumoral vessels; this differs from renal tubular cell carcinoma, which is characterized by vessel hyperplasia and a spherical shape of tumors.

### PET/CT findings

Most RCCs have a low FDG metabolism and are similar to the normal renal parenchyma. ^18^F-FDG PET has certain limitations in the detection and diagnosis of common renal cancer [24]. Because CDC is rare, there is little information on PET performance in the literature. Ye et al. reported a case of CDC with a maximum diameter of 4.6 cm in the right kidney. The SUVmax of PET was 7.0 [25]. Two patients in our group underwent PET/CT examination, and the primary lesions were highly metabolic, with SUVmax values of 14.9 and 14.3, respectively. One of the PET/CT images showed a higher metabolism in the lymph nodes, lungs, pleura, and multiple bone metastases, which was consistent with the HU and other studies [26]. Compared with other common renal cancer pathological types (such as clear cell carcinoma), CDC is characterized by high invasiveness and a poor prognosis, and it frequently shows high FDG uptake. ^18^F-FDG PET/CT is effective for the diagnosis of renal tumor metastasis. Besne et al. showed that the 5-year survival rate of patients with distant metastasis of urinary tumors is 0–20%. However, resection of isolated metastases increases the 5-year survival rate to 25–50% [27]. Therefore, early detection of metastases is essential. Safaei et al. [28] reported that the sensitivity and specificity of PET for detecting renal cell carcinoma metastases are 87% and 100%, respectively. Majhail et al. analyzed the biopsy or surgical resection samples from 36 metastatic lesions in 24 patients with RCC. The results showed that the specificity and positive predictive value of ^18^F-FDG PET/CT for distant metastasis were 100% [29]. In this study, PET/CT of the lymph nodes showed no metastasis after surgical resection. The diagnosis of lymph node metastasis by PET/CT needs to be further investigated.

### Local invasion and distant metastasis

CDC is a highly malignant tumor that often shows strong invasiveness and early metastasis [17, 30–34]. The incidence of extracranial metastasis of CDC tumors in this group supports the early metastasis of CDC. The incidence of metastasis reached 69%. The rapid metastatic spread and aggressiveness of CDC may be due to its central or perihilar location [35]. CDC is characterized by infiltration into the kidney and local lymph node metastasis, as well as distant metastasis. Most patients show lymph node enlargement and metastasis to distant organs. Lymph node metastasis accounts for 80%, lung and adrenal metastasis account for 25%, and liver metastasis accounts for 20%. The prognosis is extremely poor, and patients die within 2 years of onset. In this series, nine (69.2%) cases had lymphatic metastasis, three (23.1%) cases had bilateral lung metastasis, two (15.4%) cases had adrenal gland involvement, one (7.7%) case had inferior vena cava involvement, one (7.7%) case had bilateral pleural metastasis, one (7.7%) case had brain metastasis, one (7.7%) case had bone metastases (including the right rib, pubis, and left scapula). This is due to its high degree of malignancy and invasive biological characteristics.

The present study had several limitations. The main limitation was that the number of CDCs was too small for the CT and histopathologic analysis to be significant. Further studies with a larger number of cases are necessary.

## Conclusion

CDC has a poor prognosis and most patients develop metastatic disease. Early diagnosis is essential and may increase patient survival. According to the biological characteristics and pathology of CDC, comprehensive CT and MRI examinations, dynamic enhancement of CT, and MRI combined with multi-parameter observation and post-processing reconstruction are important to define the characteristics of the lesion and identify other kidney lesions. PET/CT examination is valuable and provides important data for designing surgical strategies and selecting the optimal treatment.

## Data Availability

The datasets generated and analyzed during the study are not publicly available due to patient privacy, but are available from the corresponding author upon reasonable request.

## Abbreviations

CDC: Collective duct carcinoma
RCC: Renal cell carcinoma
CT: Computed tomograghy
PET/CT: Positron emission tomography computed tomography
MRCP: Magnetic resonance cholangiopancreatography
^18^F-FDG: ^18^F-fluorodeoxyglucose
SUV: Standardized uptake value
